# Impact of Non-Floral Sugar Sources and Feeding Protocols on the Longevity, Reproduction, and Parasitism of *Mastrus ridens* (Hymenoptera: Ichneumonidae)

**DOI:** 10.3390/insects17070693

**Published:** 2026-07-03

**Authors:** Macarena M. Galdames, Tania Zaviezo

**Affiliations:** Facultad de Agronomía y Sistemas Naturales, Pontificia Universidad Católica de Chile, Avda. Vicuña Mackenna 4860, Macul, Santiago 7820436, Chile; macarena.galdames@uc.cl

**Keywords:** codling moth, conservation biological control, extrafloral nectar, honeydew, natural enemy

## Abstract

Successful conservation biological control programs require a good understanding the nutritional ecology of natural enemies to maximize their field efficacy. This study evaluated the effects of non-floral sugar sources on fitness parameters of *Mastrus ridens*, a specialized parasitoid of the codling moth. In the laboratory, we compared the longevity, parasitism, and reproduction of adult parasitoids exposed to different sugar sources, including *Vicia faba* extrafloral nectar, honeydew from several hemipteran species, diluted honey (positive control), and no-sugar controls. We also tested the effects of exposing parasitoids to honey for only 24 h. The results showed that sugar availability and source type significantly affected parasitoid performance. Extrafloral nectar was better than mealybug honeydew, with female offspring numbers similar to those exposed to honey, and increased the number of parasitized host larvae by up to 3.5-fold compared to no-sugar conditions. The effects of honeydew effects on male longevity were variable, possibly explained by its composition, and the mode of presentation. Parasitoids with a short exposure to high-quality sugar performed poorly. Our findings indicate that successful conservation and augmentative biological control programs using this species require field provision of high-quality, accessible sugar resources.

## Simple Summary

Conserving natural enemies in the field requires understanding their food requirements, in particularly, sugar sources for their maintenance and activity. In this study, we investigated the effects of non-floral sugar sources on *Mastrus ridens*, a beneficial wasp that attacks codling moth caterpillars. In the laboratory, we exposed wasps to the extrafloral nectar of broad beans, the sugary secretion (honeydew) of several sap-feeding insects, and positive (honey) and negative (no sugar) controls. We also tested the effects of a short 24 h exposure to honey. Our results revealed that not all sugar sources were equally beneficial for the wasps. Broad bean extrafloral nectar was better than mealybug honeydew because the wasps reproduced as much as with honey and increased their attack on the pest caterpillars by up to 3.5 times compared to no sugar control. Honeydew effects were variable among males, likely due to differences in composition and mode of presentation. Wasps exposed to honey for a short period performed poorly. Our findings provide valuable information for the use of this natural enemy and underscore the importance of having high-quality, accessible sugar resources in the field to enhance its impact. This will help in designing effective biodiversity-rich orchards with fewer chemical sprays.

## 1. Introduction

Most adult hymenopteran parasitoids require sugar meals, as they depend primarily or solely on carbohydrates as a source of energy for their maintenance and activity [1,2]. In the laboratory, the longevity and fecundity of parasitoids increase markedly when they receive sugar meals, usually diluted honey (e.g., [3,4,5,6,7,8,9]). In the field, floral nectar is an important sugar source [10,11,12], but it may not be the only or most important source in agroecosystems [13]. Extrafloral nectar (EFN) is another plant-derived sugar source secreted by organs outside flowers, which many plants have evolved to attract herbivores’ natural enemies, and its sugar composition is often similar to that of floral nectar [3,14,15]. Some advantages of this sugar source over floral nectar are that it is often available for longer periods of time (i.e., before, during, and after flowering) and is more accessible because flower architecture does not interfere with the insect’s path to the nectar [14,16,17]. Nevertheless, their numbers and secretion can be influenced by several factors, such as plant nutritional status, plant age, environmental conditions, or damage, as documented in faba beans (*Vicia faba* Linnaeus, 1753; Fabaceae) [18,19,20]. This plant species is frequently used in the context of extrafloral nectar provisioning as a strategy for conservation biological control [20,21,22,23].

Another alternative sugar source in the field is honeydew secreted by many hemipteran insects that feed on phloem and xylem. Some of these insects are common pests or occur in non-crop vegetation in or around fields, including aphids, mealybugs, whiteflies, and soft scale insects [14,24,25,26,27]. An advantage of honeydew over floral nectar is that it can be present in large quantities for extended periods within a season [13,14,28]. In addition, honeydew accessibility is not mediated by flower architecture. However, in some cases, it can be limited by its viscosity or the presence of competitors, such as ants [13,29,30]. Similar to floral and extrafloral nectar, the composition and quantity of honeydew can be influenced by several factors, particularly the insect species and its developmental stage, the plant species and its nutritional status, environmental conditions, and time since deposition, among others [26,27,31,32,33,34,35]. In general, honeydew is regarded as a lower-quality sugar source [36,37]. Studies with parasitoids have focused on those that parasitize honeydew producers, where an evolutionary history might be involved [38], and only few studies have focused on parasitoids that attack pests that do not produce honeydew (see the studies compiled by [25]).

Apples are an important fruit crop in temperate regions, representing the most -produced temperate fruit worldwide and ranking among the top three fruits produced in 2025 [39,40]. Organic apples are also a growing trend, supported by consumer preference for pesticide-free produce [41], consequently promoting biological control. In Chile, there are 20,000 ha (including conventional and organic), and it currently represents the 5th most-planted fruit crop in the country and is second in terms of volume produced [42]. In many apple orchards, cover crops between tree rows are established as a complementary measure to manage soil fertility, prevent erosion, and suppress weed growth. Still, they could also contribute to pest control by providing complementary resources for natural enemies (i.e., nectar, pollen, alternative prey) [43]. Legumes (Fabaceae), including *V. faba* and other *Vicia* species, are often used in cover crop mixes because they can aid in fertilization through nitrogen fixation from the atmosphere into the soil [44,45,46]. Moreover, *Vicia* species are known to have extrafloral nectaries on the stipules at the base of leaves [47].

Apple plants are also hosts to many honeydew-producing pests, with aphids and mealybugs being among the most common worldwide [48,49,50]. However, the most important apple pest is the codling moth, *Cydia pomonella* (L., 1758) (Lepidoptera: Tortricidae), because its larvae feed inside apples (fruit tunneling), rendering the fruit unmarketable [51,52]. *Mastrus ridens* Horstmann 2009 (Hymenoptera: Ichneumonidae) is an important parasitoid from the same region as the pest and attacks mature codling moth larvae in their cocoons. This parasitoid has been imported to several countries, but variable results after its release in different regions have been reported, which might be related to intrinsic factors, such as the low genetic diversity of released populations [53,54,55], or extrinsic factors, such as climate or the availability of sugar sources in commercial orchards. This last resource could be critical for its establishment and impact, as *M. ridens* survival can increase by 5 to 8-fold in the laboratory when fed diluted honey (e.g., [5,8]).

In previous studies in Chile, of the 15 native and non-native plant species evaluated as sugar sources for *M. ridens*, only three showed promise for conservation biological control. A major limitation was nectar accessibility, particularly among Asteraceae species [17]. The impact of other sugar sources on *M. ridens* longevity and reproduction has not been studied. Identifying non-floral sugar sources in apple orchards is essential for providing *M. ridens* with a more accessible complementary food source. However, the suitability of extrafloral nectar and honeydew as dietary supplements varies widely, mainly due to differences in sugar profiles and amino acid content. Therefore, targeted evaluations are important before making system-specific recommendations for biocontrol. We hypothesized that EFN benefits parasitoids more than honeydew, with varying efficacy across hemipteran species. The objective of this study was to determine the effects of extrafloral nectar from *V. faba* and honeydew from different hemipteran insects on the longevity, parasitism, and reproduction *M. ridens*.

## 2. Materials and Methods

### 2.1. Parasitoid and Host Origin and Handling

The parasitoid and host specimens used in the experiments originated from established laboratory colonies at the Facultad de Agronomía y Sistemas Naturales of Pontificia Universidad Católica de Chile, Santiago, Chile. The host *Cydia pomonella* was originally collected from apple orchards in central Chile in 2011 and has been maintained in the laboratory using artificial diets (mainly Stonefly Heliothis Diet; Ward’s Natural Science, Rochester, NY, USA) for larval feeding. Once larvae were fully developed (approximately 14 days), they were removed from the diet and provided with strips of corrugated cardboard (1.5 cm wide) as a substrate for spinning their cocoon. The 5th-instar cocooned larvae were either allowed to continue their development to maintain the moth colony or exposed to parasitoids as hosts.

The current *Mastrus ridens* laboratory colony is a mixture of individuals collected in Kazakhstan (Almaty region) in 2013 and 2015, and others brought to our facility in 2014 from a New Zealand mass-rearing colony (see [53] for details). The parasitoid individuals used in the experiments came from populations reared under laboratory conditions (25 ± 2 °C, 40 ± 10% RH, and 16 h light:8 h dark photoperiod) for approximately 110 generations, in cages made of woven nylon netting (BugDorm-4F3030, MegaView Science Co., Ltd., Taichung, Taiwan), provided with water and honey solution. Strips of corrugated cardboard containing cocooned codling moth larvae were regularly introduced into these cages. For the experiments, parasitoid cocoons were collected from cardboard exposed in the cages for 2 weeks and placed in vials until adult emergence.

### 2.2. Sugar Sources and Presentation Mode

As sugar food for adult parasitoids, we used extrafloral nectar from *V. faba* and the honeydew of four hemipteran insects found feeding in or around apple orchards in Chile. These were the citrophilus mealybug (*Pseudococcus calceolariae* Maskell, 1879, Pseudococcidae), woolly apple aphid (*Eriosoma lanigerum* (Hausmann, 1802), Aphididae), spirea aphid (*Aphis spiraecola* Patch, 1914, Aphididae), and oleander aphid (*Aphis nerii* Boyer de Fonscolombe, 1841, Aphididae).

Following the protocol of [56], *P. calceolariae* honeydew was extracted from colonies maintained on butternut squash in our laboratory. Briefly, parafilm^®^ pieces were placed beneath a severely infested butternut squash for 48 h, then examined with a magnifying glass for the presence of abundant honeydew. The parafilm was cut into 2.5 × 6 cm pieces for use in the experiments. Some of these pieces were stored in sealed plastic bags at −20 °C to ensure a constant supply. Honeydew from aphids was sourced directly from infested plants in the campus gardens in Santiago or from the agricultural experimental station at Pirque (25 km to the south). For *E. lanigerum*, severely infested apple shoots were collected and cut into 5 cm-long pieces for the experiment. For *A. spiraecola,* shoots of *Spiraea cantoniensis* Lour (Rosaceae) with infested leaves were collected and cut into 8 cm long pieces, and for *A. nerii*, infested leaves of *Nerium oleander* L. (Apocynaceae) were collected. For the extrafloral nectar (EFN), *V. faba* seeds were sown in 3 L pots 6–9 weeks before the experiments and fertilized in the third week after planting with 10 g of germinal fertilizer (7:18:20% N:P:K, Anasac, Santiago, Chile). Two weeks before the experiment, the plants were subjected to severe foliar damage by removing one-third of each leaf to stimulate extrafloral nectary development [18].

Additionally, diluted organic honey (50% *v*/*v*) was used as a control, provided either continuously or exclusively for the first 24 h. The latter simulates a scenario in which mass-reared parasitoids are fed only pre-release and have no subsequent access to sugars.

### 2.3. Male Longevity with Different Food Sources

To evaluate diet effects, recently emerged (<24 h), unfed adult male *M. ridens* were placed individually into 250 mL plastic containers and randomly assigned to one of six sugar treatments: citrophilus mealybug honeydew collected on Parafilm (*n* = 15); woolly apple aphid honeydew on infested apple shoots (*n* = 15); spirea aphid honeydew on infested shoots and leaves (*n* = 7); oleander aphid honeydew on infested leaves (*n* = 4); diluted honey painted on container walls using a fine brush (*n* = 14); and diluted honey placed on a Parafilm strip, which was removed after 24 h (*n* = 15). To maintain constant access to sugar meals, except for the 24 h restricted honey treatment, infested plant material was replaced twice per week, and honey and parafilm pieces with honeydew were replenished three times per week. In all these treatments, access to water was provided throughout the experimental period via moistened cotton balls placed inside the containers, which were replenished three times per week. Finally, two negative controls (no sugar) were included: a water-only control (*n* = 16), in which individuals received only the moistened cotton ball setup, and an absolute control (*n* = 15) with no sugar or water. This absolute control was included to determine the specific effect of water availability on the longevity of parasitoid.

For the extrafloral nectar (EFN) treatment (*n* = 14), newly emerged *M. ridens* adult males were individually confined in organza bags (approximately 20 × 15 cm) and placed on *V. faba* shoots containing at least four stipules with active nectaries. Experimental units were monitored daily and transferred to new vegetative shoots when flower buds appeared to prevent exposure to floral nectar.

Experiments were carried out between April and July 2024, according to the availability of insects and sugar sources, in a growth chamber at 25 °C, 60% RH, and a 16 h light: 8 h dark photoperiod, except for those conducted with faba bean shoots. The latter were maintained in a greenhouse under similar but more variable conditions.

Longevity was recorded through daily inspections of the experimental units. Following death, male specimens were collected to measure hind tibia length as a proxy for size, using a Leica^®^ microscope (Leica Microsystems, Singapore, Singapore) (LAS EZ^®^ imaging software version 3.4.0), and analyzed using ImageJ version 1.54g [57].

### 2.4. Female Longevity, Parasitism, and Reproductive Output with Different Food Sources

Because we had less availability of females and sugar sources, these experiments were carried out with a subset of the treatments used for the males: citrophilus mealybug honeydew (*n* = 15), extrafloral nectar (EFN) (*n* = 16), diluted honey replenished twice a week (*n* = 12), diluted honey for 24 h (*n* = 15), water (*n* = 12), and absolute control with no sugar or water sources (*n* = 7).

The experimental procedure for females followed the protocol used for males, except that a male was initially introduced into each experimental unit for 24 h to allow for mating. Additionally, to assess parasitism and reproduction, females were subsequently provided with three cocooned codling moth larvae on small corrugated cardboard strips (1.5 × 0.5 cm). Three additional larvae were introduced every 3–4 days to maintain a supply of six host larvae per female, per week. Each cardboard strip remained in the experimental unit for 6–7 days before being removed, labeled, and incubated at 25 °C. After 13–14 days of initial exposure to the parasitoid, host larvae were carefully examined to determine parasitism while ensuring the integrity of the immature developing parasitoids. Parasitized hosts were kept until adult emergence to record the number and sex of progeny.

Experiments were carried out between May and July 2024 under the same laboratory conditions as the male experiments (rearing chamber at 25 °C, 60% RH, 16 h light: 8 h dark photoperiod), and in this case, the *V. faba* plants were also kept in the rearing chambers. As with males, longevity was recorded through daily inspections, and following death, specimens were collected to measure their hind tibia length.

### 2.5. Data Analysis

Initially, the data were analyzed for normality and homogeneity of variance using the Shapiro–Wilk and Levene’s tests, respectively. As none of the variables, except female tibia length, met the assumptions of parametric analyses, we used Generalized Linear Models (GLMs). The effects of food sources on female and male longevity were analyzed using a GLM with a negative binomial distribution to correct for overdispersion (deviance ratio) in the initial Poisson model with a log link function. These models were also run with tibia length as a covariate. However, because its effects were non-significant (*p* > 0.05) and the AIC values were very similar to those of the models without the tibia, we opted for the simpler model. Differences between means were analyzed using Fisher’s LSD test (*p* < 0.05) with the Benjamini–Hochberg correction to control the false discovery rate [58]. Male and female survival was also analyzed using Kaplan–Meier curves and compared using the log-rank test. Pairwise comparisons among food-source treatments were performed using the log-rank test, adjusted for multiple comparisons [59]. Additionally, we assessed whether male and female sizes (tibia length) differed among treatments using ANOVA for females and the Kruskal–Wallis rank-sum test for males. Finally, to explore the potential effects of size (tibia length) on longevity within food sources, Spearman correlations were run independently for each treatment.

The effects of food sources on parasitism and female reproduction were studied using GLMs with a negative binomial distribution and log link function for the total number of larvae parasitized, number of larvae parasitized in the first week, lifetime adult progeny produced by a female, and clutch size (i.e., total number of progeny per parasitized larvae). The sex ratio (proportion of males in the progeny, total males/total progeny) was analyzed using a binomial distribution adjusted for overdispersion (quasibinomial) with a logit link function. Because 38% of the cases with effective reproduction (at least one progeny produced) had no female progeny, indicating a potential failure in mating, sex ratio analyses were run again, only with those cases with at least one female progeny (*n* = 32). All GLMs were tested using a Likelihood Ratio Test (“Chisq” test for models with the binomial and negative binomial error distributions, and the “F” test for quasibinomial). Finally, Spearman correlation was used to explore the relationship among female longevity, number of larvae parasitized, and total progeny produced.

All statistical analyses were performed using R version 4.4.3 [60] and RStudio version 2025.05.01 [61]. Means presented in figures and text are adjusted means of untransformed data ± standard error.

## 3. Results

### 3.1. Effect of Food Sources and Size on Male Longevity

**Figure 1:**
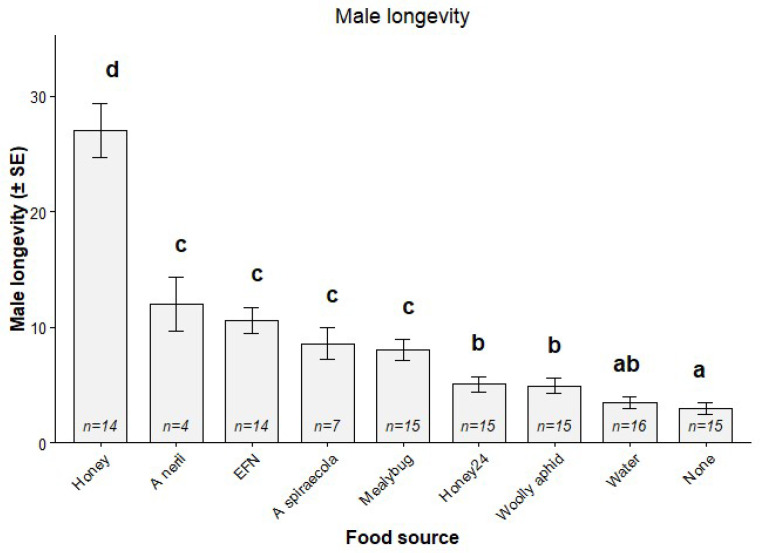
Mean (+SE) longevity (days) of *M. ridens* males when they had access to different food sources, including constant access to diluted honey (Honey) or only for 24 h (Honey24), *V. faba* plants with EFN, plant tissues with the honeydew of different species of aphids (*A. nerii*, *A. spiraecola*, Woolly aphid), parafilm pieces with mealybug honeydew (Mealybug), only water (Water) or no water and sugar sources (None). Different letters above bars indicate significant differences among treatments (LSD with Benjamini–Hochberg correction, *p* < 0.05). Numbers at the bottom of the bars indicate the number of replicates.

The longevity of *Mastrus ridens* males was significantly influenced by food source and of presentation (χ^2^ = 330.6; *df* = 8; *p* < 0.0001; Figure 1). Males lived the longest with constant access to honey (27.1 ± 2.3 d), and the least with no access to sugar and water (2.9 ± 0.5 d), or with access to water only (3.4 ± 0.5 d) (Figure 1). When males had access to plant tissues with the honeydew of the aphids *Aphis nerii* and *Aphis spiraecola*, parafilm with the honeydew of the mealybug (*P. calceoraliae*), or plants with EFN, they lived between 8 and 12 days. When they had access to honey for the first 24 h, or to shoots with woolly aphids, they lived only about 5 d. Males readily approached the honeydew under the different treatments and were observed feeding.

**Figure 2:**
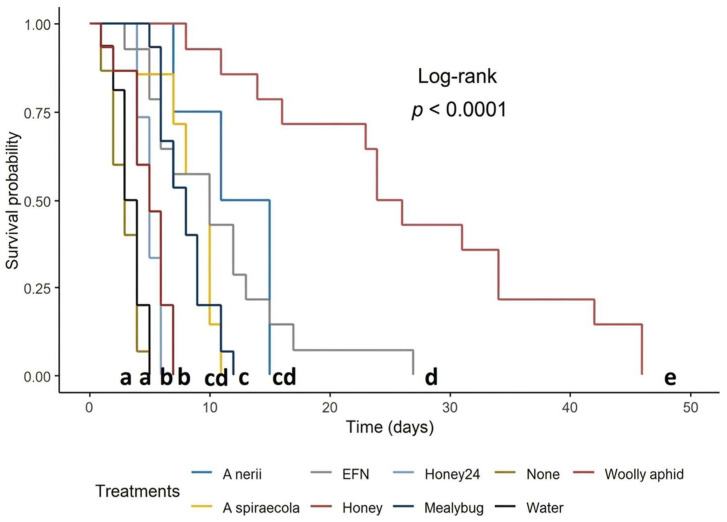
Kaplan–Meier survival curves for males with access to different food sources. Constant access to diluted honey (Honey) or only for 24 h (Honey24), *V. faba* plants with EFN, plant tissues with the honeydew of different species of aphids (*A. nerii*, *A. spiraecola*, and Woolly aphid), parafilm pieces with mealybug honeydew (Mealybug), only water (Water), or no water and sugar sources (None). Different letters at the bottom of the lines indicate significant differences among treatments (log-rank test with Benjamini–Hochberg correction, *p* < 0.05).

Survival analyses also showed significant effects of food sources and mode of presentation (χ^2^ = 142; *df* = 8; *p* < 0.0001), and differences among treatments were similar to the GLM analyses, although in the survival analysis, the woolly aphid treatment was significantly better than water, and the *A. nerii* honeydew was significantly better than the mealybug honeydew (Figure 2).

Male size was not significantly different among treatments (χ^2^ = 4.805; *df* = 8; *p* = 0.78), with mean hind tibia length varying between 1.24 ± 0.04 mm and 1.40 ± 0.08 mm. Within treatments, tibia length was not correlated with longevity (*p* values between 0.11 and 0.88), except in the treatments where males had access to honey for 24 h (*r* = 0.52; *p* = 0.045) (Figure S1).

### 3.2. Effect of Food Sources and Size on Female Longevity

**Figure 3:**
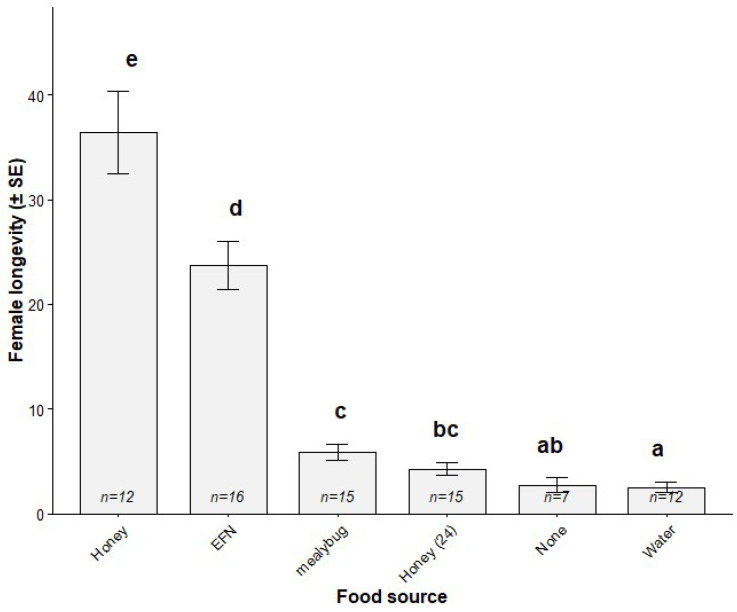
Mean (+SE) longevity (days) of *M. ridens* females when they had access to different food sources, including constant access to diluted honey (Honey) or only for 24 h (Honey(24)), *V. faba* plants with EFN, parafilm pieces with mealybug honeydew (mealybug), only water (Water), or no water and sugar sources (None). Different letters above bars indicate significant differences among treatments (LSD with Benjamini–Hochberg correction, *p* < 0.05). Numbers at the bottom of the bars indicate the number of replicates.

The longevity of *M. ridens* females was significantly influenced by the food source (χ^2^ = 369.2; *df* = 5; *p* < 0.0001; Figure 3). Similar to males, females also lived the longest with constant access to honey (36.4 ± 3.9 d), and the least with access to water only (2.5 ± 0.5 d) or no access to sugar and water (2.7 ± 0.7 d) (Figure 3). The second-best longevity was observed when females had access to EFN (23.8 ± 2.3 d), which differed from all other treatments. Females lived for less than six days when they had access to mealybug honeydew or honey for 24 h (Figure 3).

**Figure 4:**
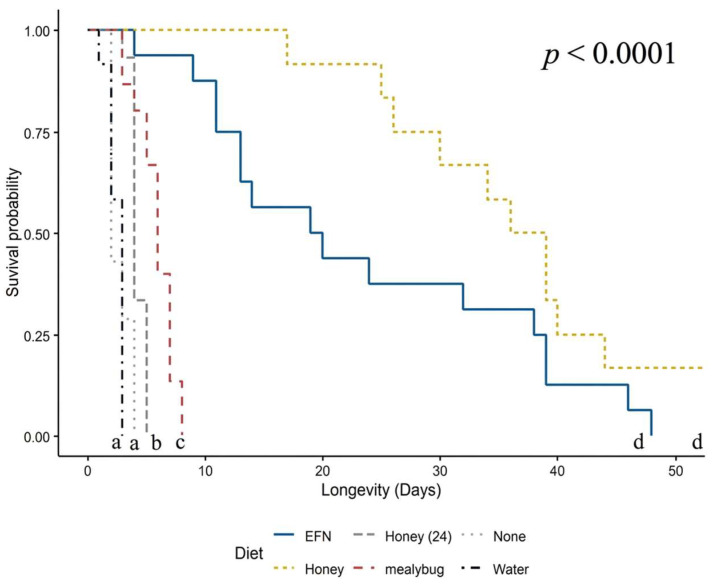
Kaplan–Meier survival curves for females with access to different food sources. Constant access to diluted honey (Honey) or only for 24 h (Honey(24)), *V. faba* plants with EFN, parafilm pieces with mealybug honeydew (mealybug), only water (Water), or no water and sugar sources (None). Different letters at the bottom of the lines indicate significant differences among treatments (log-rank test with Benjamini–Hochberg correction, *p* < 0.05).

Survival analyses also showed significant effects of food sources (χ^2^ = 117; *df* = 5; *p* < 0.0001), with honey and ENF being statistically similar, followed by mealybug honeydew, and then honey for 24 h, which was better than water and no food (Figure 4).

Female size was not significantly different among treatments (F = 0.95; *df* = 5; *p* = 0.46), with mean hind tibia length varying between 1.33 ± 0.04 and 1.43 ± 0.04 mm. Within treatments, tibia length was correlated with longevity for the treatments without sugar access, that is, water (*r* = 0.66; *p* = 0.019) and no sugar and water (*r* = 0.90; *p* = 0.006), but no significant correlations were found for the other four treatments (*p* between 0.14–0.93) (Figure S2).

### 3.3. Effect of Food Sources on Parasitism and Female Reproductive Output

**Figure 5:**
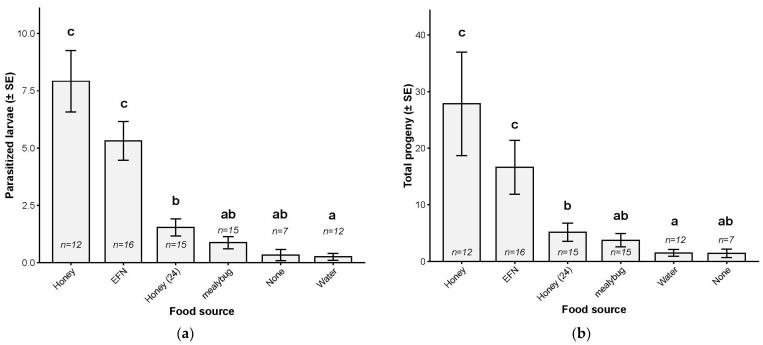
(**a**) Mean (+SE) larvae parasitized by *M. ridens* females in their lifetime. (**b**) Mean (+SE) lifetime reproductive output (adult offspring emerged) of *M. ridens* females when they had access to different food sources, including constant access to diluted honey (Honey) or only for 24 h (Honey(24)), *V. faba* plants with EFN, parafilm pieces with mealybug honeydew (mealybug), only water (Water), or no water and sugar sources (None). Different letters above bars indicate significant differences among treatments (LSD with Benjamini–Hochberg correction, *p* < 0.05). Numbers at the bottom of the bars indicate the number of replicates.

The number of larvae parasitized by a female varied significantly according to the food source (χ^2^ = 131.6; *df* = 5; *p* < 0.0001; Figure 5a). Females parasitized most larvae when they had constant access to honey or EFN (7.9 ± 1.3 and 5.3 ± 0.8 larvae, respectively). When females had access to honey for 24 h, they parasitized only 1.5 ± 0.4 larvae throughout their lifetime. With mealybug honeydew or no access to sugar (water and no food), they parasitized fewer than one larva in their lifetime (Figure 5a). The number of parasitized larvae decreased linearly from approximately 3 and 2.5 in week one for honey or EFN, respectively, to 0 in week six (Figure S3). In the other treatments, females parasitized larvae only in week 1. The number of larvae parasitized by a female in the first week also varied significantly according to food source (χ^2^ = 54.32; *df* = 5; *p* < 0.001), following the same trend as the total number of parasitized larvae (Figure S4).

Females’ total reproductive output (total adult offspring emerged) also varied significantly according to food source (χ^2^ = 56.3; *df* = 5; *p* < 0.0001; Figure 5b). Similar to parasitism, most offspring were produced when females had constant access to honey or EFN (27.8 ± 9.1 and 16.6 ± 4.8 offspring, respectively). When females had access to honey for 24 h, they had 5.1 ± 1.6 offspring, and with mealybug honeydew, they had 3.7 ± 1.2 offspring. When females had no access to sugar (water only, no food), they had fewer than 2 offspring on average in their lifetimes. Most females in these treatments did not live long enough to parasitize larvae. In fact, longevity, number of parasitized larvae, and total reproductive output were all significantly and strongly correlated (*p* < 0.0001; *r* > 0.75 for all combinations).

### 3.4. Effect of Food Sources on Clutch Size and Offspring Sex Ratio

**Figure 6:**
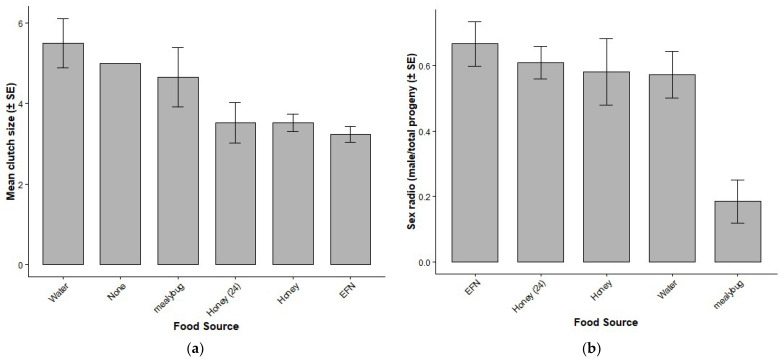
(**a**) Mean clutch size (total offspring/total parasitized larvae) (+SE) and; (**b**) sex ratio (males/total progeny, considering only clutches with at least one female) for *M. ridens* females when they had access to different food sources, including constant access to diluted honey (Honey) or only for 24 h (Honey(24)), *V. faba* plants with EFN, parafilm pieces with mealybug honeydew (mealybug), only water (Water), or no water and sugar sources (None).

The mean clutch size (total offspring/total parasitized larvae) varied between 3.2 ± 0.2 and 5.5 ± 1.1 offspring per larva but did not vary significantly according to food source (χ^2^ = 9.5; *df* = 5; *p* = 0.09; Figure 6a). When the sex ratio was calculated using the entire dataset (offspring > 0, *n* = 50), the proportion of males in the offspring varied from 1 ± 0 (only one parasitized larva with only male offspring in the no water and sugar treatment) to 0.46 ± 0.13, but there was no significant effect of food source (F = 1.5; *df* = 5; *p* = 0.22). In the data, where at least one female offspring was produced (female offspring > 0, *n* = 32), the proportion of males in the offspring varied from 0.66 ± 0.06 to 0.18 ± 0.06 (Figure 6b), with no significant effects of food source (F = 1.9; *df* = 4; *p* = 0.14).

## 4. Discussion

This study evaluated non-floral sugar sources for *Mastrus ridens*, a codling moth parasitoid introduced to several countries. Its unexpectedly low impact in some of these regions may stem from the limited availability of sugar for adults in the field.

The longevity of both males and females of *M. ridens* was greater when they were exposed to diluted honey, as expected, owing to the types of sugars it contains, their high concentration, and ready access; thus, it was considered a “positive control”. The extrafloral nectar of *Vicia faba* emerged as an interesting potential sugar source, as females had high longevity, parasitism, and progeny production relative to no-sugar controls, and EFN performed similarly to honey for parasitism and total progeny. Males also had higher longevity with EFN than no-sugar controls, even though the more variable conditions compared to the other treatments, potentially affected their performance. As extrafloral nectar is more accessible and available throughout the year, and *V. faba* has shown positive effects in some agroecosystems [14,62], further field testing of *M. ridens* is warranted. Using this plant species as a cover crop in fruit orchards is also attractive because it can provide multiple benefits beyond promoting biological control [63]. However, it is also important to evaluate whether it can serve as a food source for pest species present in crops [12] or for other arthropods, such as ants. The sugar provided to ants by EFN can have a positive [30,64] or negative [65,66] impact on parasitoids present in the fields. Therefore, before recommending the use of plants with EFN in conservation biological control, it is important to conduct field studies to evaluate the overall impact on the most relevant species interacting within the crop and to integrate both costs and benefits [67].

Females were also exposed to the honeydew of mealybugs, and their longevity, parasitism, and total progeny were much lower than those fed on honey and EFN. Other studies have shown that honeydew is usually a lower-quality food source than honey or flower nectar [4,29,68,69,70]. However, some studies have reported variable benefits [13,29,56,71,72,73], while others have shown that honeydew is as good as honey or specific sugars [74,75,76]. This variability in effects underscores the importance of conducting studies on the target species before making recommendations.

In males, we tested several hemipteran honeydews and observed varying longevity depending on the species and mode of presentation. Males had shorter longevity when fed on the honeydew of the woolly aphid, *Erisoma lanigerum*, than on the honeydew of the other aphids or the mealybug. This is probably explained by the lower accessibility of woolly aphid honeydew compared with the others, because it is coated with a wax layer and has high viscosity, which presents a difficulty even for the specialist woolly aphid parasitoid [13]. Additionally, in our experimental setup, *M. ridens* was exposed to apple tree shoots infested with *E. lanigerum* in the laboratory. We observed that the woolly wax secretion gradually covered the insects, ultimately compromising their mobility and ability to fly (Figure S5). Male longevity when exposed to the honeydew of the other species was similar and not significantly different, which was unexpected, as other studies have shown the effects of aphid species and the plants they feed upon [71,72,77].

The lack of differences in male longevity when exposed to accessible aphid honeydew and the low performance on mealybug honeydew should be interpreted with caution because we had few replicates for some aphid treatments. In addition, the feeding substrate and exposure methodology differed, particularly between aphids and mealybugs. These were fed on fruit (squash), and honeydew was collected on parafilm pieces. In contrast, the aphids were fed on shoots and foliage and were introduced into the experimental arenas as colonies. This may have resulted in less mealybug honeydew and/or lower sugar content, as they were not directly feeding on the phloem. It would be interesting to compare these results with those of experiments using mealybug colonies feeding on shoots and foliage. Other factors might also have differed among the exposure modes (e.g., surface structure, plant tissue effects, plant volatiles, waxes, and shelter), which in our study confounded the effects of sugar quality with the physical and biological factors related to how the sugars are presented. Therefore, in future research, it would be valuable to measure the honeydew deposition volume and its specific chemical and physical properties to provide a deeper mechanistic understanding of its effects on parasitoid fitness.

In this study, we also evaluated the short supply of high-quality sugars (honey24) to determine whether pre-release sugar feeding alone, within an augmentative biological control program, could provide a significant benefit. Nevertheless, this treatment performed very poorly, with male and female longevity, parasitism, and total progeny similar to those of the negative controls (water or no food). This poor performance is probably explained by the fact that *M. ridens* is synovogenic (i.e., continues to mature eggs during its adult life) and that parasitizing the late-instar larvae of its host, which includes venom injection for paralysis and a long handling time, is energetically costly [5,55]. In the field, *M. ridens* longevity, parasitism, and progeny should be even lower, given that in the laboratory, host availability was relatively high, whereas in the field, host encounter rates may be much lower, and females would have to spend energy searching for hosts [78]. Consistent with previous findings [55], laboratory studies indicate that in *M. ridens*, both parasitism and progeny production are concentrated during the initial two weeks, despite the continuous access to honey. In our study, differences in parasitism between the best-performing treatments (honey and EFN) and the mealybug honeydew and no-sugar treatments were observed as early as the first week and increased when considering the total lifetime fecundity. In the first week, the 24 h honey treatment was not different from the EFN treatment, but when considering lifetime parasitism, it was much worse. Thus, a high-quality, readily available sugar source in the field could improve biological control by extending the female lifespan and increasing the per-day attack capacity.

Even when food sources did not affect other clutch size (number of progeny produced by parasitized larvae) and sex ratio (proportion of males in the progeny), there was a trend of larger clutch size in the no sugar treatments (negative controls) compared to the better sugar source treatments (honey and EFN) (*p* = 0.09). This suggests that oviposition decisions in *M. ridens* are influenced by the female’s energy state and life expectancy, with females laying fewer eggs on a host when they have sufficient energy reserves to live long enough to find and parasitize another host. These results contradict those of Bezemer and Mills [78], possibly because of the measured variables (adult progeny emerged vs. eggs laid). On the other hand, our results align with the optimal clutch-size theory (i.e., maximizing lifetime fitness gain rather than the gain to an individual host) and dynamic, state-dependent decisions [79,80,81,82,83,84,85]. In the case of sex ratio, previous studies on *M. ridens* have not found effects of food provision, host size, or female size on sex ratio [55,78].

Finally, it is well known that there is a positive relationship between body size and longevity in many parasitoid species [86]. Nevertheless, in this study, male *M. ridens* size did not affect longevity, whereas in females, significant correlations were found only in the no-sugar treatments (water and no food). Bezemer et al. [5] found similar results for the same species: larger females lived longer when they had no access to honey, regardless of host presence. In honey-fed females, larger females lived longer in the absence of hosts, whereas no relationship was observed when hosts were present (as in our study). However, larger females consistently attacked more larvae and produced more progeny. This indicates that with abundant food and hosts, larger females prioritize allocating resources to reproduction over maintenance [5].

## 5. Conclusions

The results of this study confirm the importance of sugar sources for the fitness of *Mastrus ridens* and its potential impact on its host, the codling moth. However, the positive effects of different sugar sources depend on their characteristics, most probably their sugar composition and accessibility, and presentation mode, which deserve further investigation. Among the sugar sources tested, the EFN of *Vicia faba* was promising: females achieved longevity and parasitism similar to those in diluted honey, making it a good candidate for future field evaluation. In contrast females did not perform well on *Pseudococcus calceolariae* honeydew. Males tested on additional honeydew sources showed similar longevity with honeydew from *Aphis nerii* and *Aphis spiraecola*, and with EFN. Future research should investigate the effects of honeydew from these and other aphid species on female fitness, which is most relevant to biological control, as well as how their chemical composition influences the nutritional suitability for *M. ridens*. While laboratory assays are crucial for identifying promising sugar sources, subsequent field evaluations are essential to account for complex ecological interactions and abiotic conditions within the orchards. From a practical standpoint, our findings also have implications for augmentative biological control programs using *M. ridens*, because feeding adults high-quality sugar before release is not enough; they also need accessible sugar sources in the field to sustain their survival and reproduction. More broadly, understanding how non-crop resources benefit natural enemies and their role in multitrophic interactions will contribute to the design of diversified, sustainable agroecosystems through targeted habitat management [87].

## Data Availability

The original data presented in the study are openly available in FigShare at 10.6084/m9.figshare.32521131.

## Supplementary Materials

The following supporting information can be downloaded at: https://www.mdpi.com/article/10.3390/insects17070693/s1. Figure S1: Relationship between male size (hind tibia length) and longevity by food source treatment; Figure S2: Relationship between female size (hind tibia length) and longevity by food source treatment; Figure S3: Number of parasitized larvae per week by *M. ridens* females when they had access to different food sources; Figure S4: Number of parasitized larvae in the first week by *M. ridens* females when they had access to different food sources; Figure S5: *Mastrus ridens* males foraging in colonies of *E. lanigerum*, and deposition of wax on its bodies limiting its mobility.
