# Supplementary material for: Impact of Non-Floral Sugar Sources and Feeding Protocols on the Longevity, Reproduction, and Parasitism of Mastrus ridens (Hymenoptera: Ichneumonidae)
Source: Insects. 2026 Jul 3;17(7):693. doi: 10.3390/insects17070693 (PMC13411347; doi:10.3390/insects17070693)

insects

MDPI

Supplementary material

Impact of Non-Floral Sugar Sources and Feeding Protocols on the Longevity, Reproduction, and Parasitism of *Mastrus ridens* (Hymenoptera: Ichneumonidae)

Macarena M. Galdames and Tania Zaviezo *

Figure S1. Relationship between male size (hind tibia length) and longevity by food source treatment. The table below shows the correlation coefficient and the p-value.

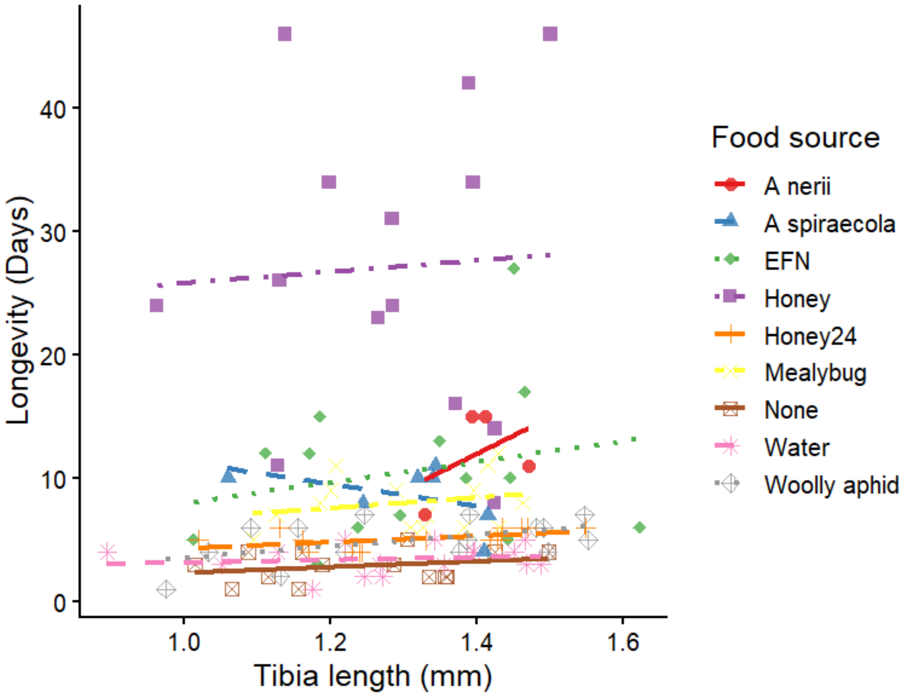

	Foodsource	Rho_Spearman	*p*_value
1	A nerii	0.316	0.684
2	A spiraecola	-0.408	0.364
3	EFN	0.258	0.373
4	Honey	0.044_	0.881
5	Honey24	0.524	0.0449
6	Mealybug	0.209	0.456
7	None	0.260	0.369
8	Water	0.131	0.629
9	Woolly aphid	0.435	0.105

Figure S2. Relationship between female size (hind tibia length) and longevity by food source treatment. The table below shows the correlation coefficient and the p-value.

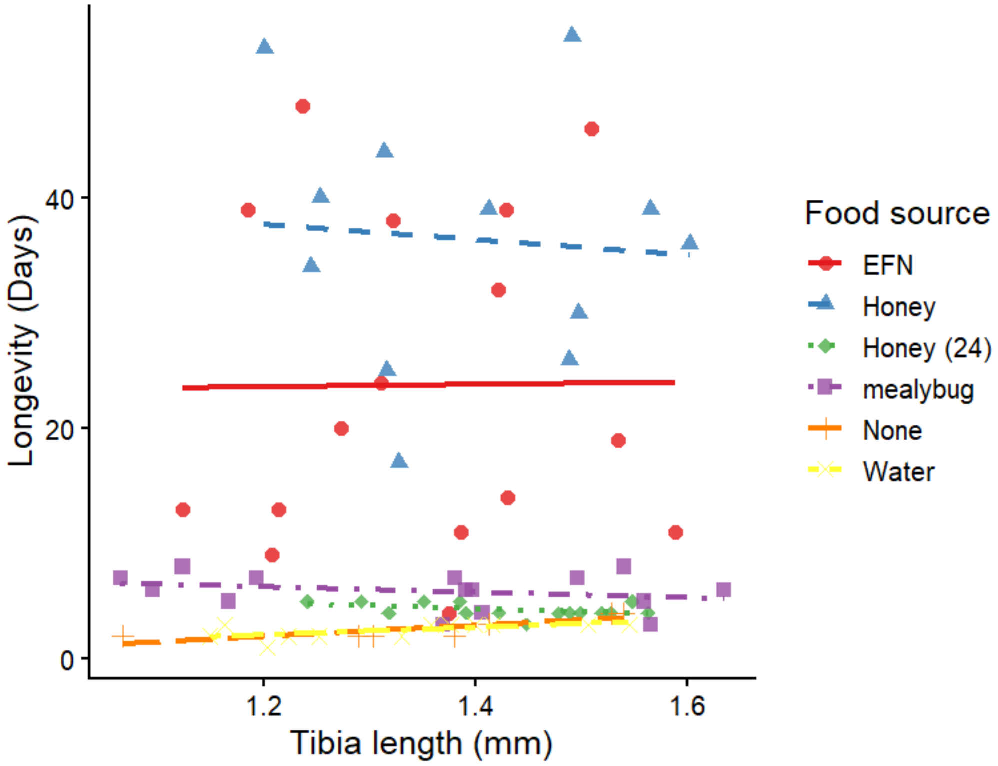

	Foodsource	Rho_Spearman	*p*_value
1	EFN	0.025	0.927
2	Honey	-0.168	0.601
3	Honey (24)	-0.404	0.135
4	mealybug	-0.274	0.324
5	None	0.896	0.006
6	Water	0.662	0.019

Figure S3. Number of parasitized larvae per week by *M. ridens* females when they had access to different food sources, including constant access to diluted honey (Honey) or only for 24 h (Honey(24)), *V. faba* plants with EFN, parafilm pieces with mealybug honeydew (mealybug), only water (Water), or no water and sugar sources (None). The Honey(24), Water, and None only presented parasitism in week one.

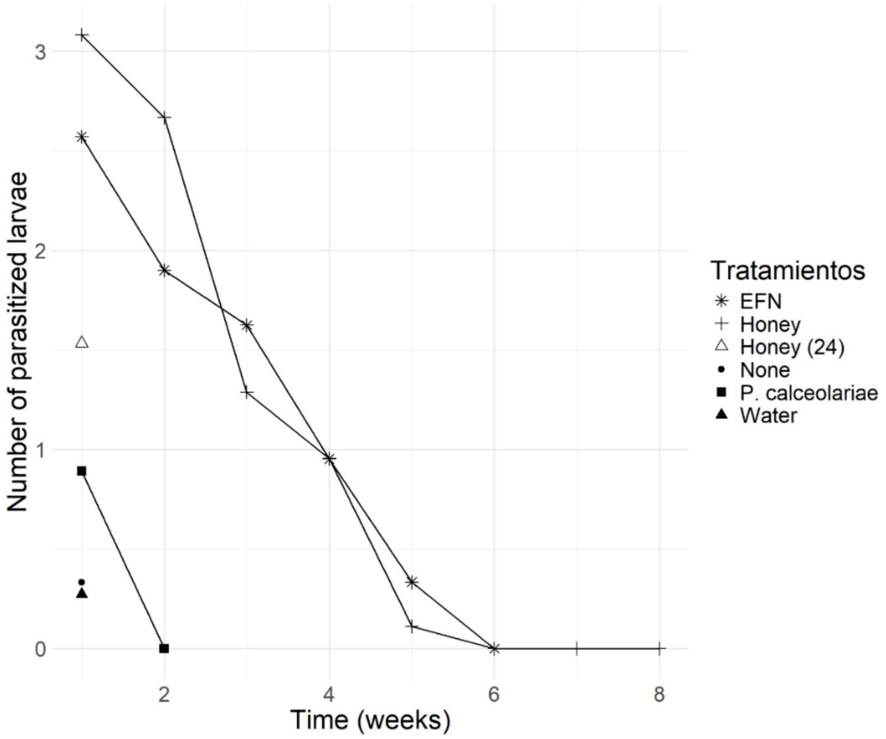

Figure S4. Number of parasitized larvae in the first week by *M. ridens* females when they had access to different food sources, including constant access to diluted honey (Honey) or only for 24 h (Honey(24)), *V. faba* plants with EFN, parafilm pieces with mealybug honeydew (mealybug), only water (Water), or no water and sugar sources (None). Different letters above bars indicate significant differences among treatments (LSD with Benja-mini-Hochberg correction, $p < 0.05$).

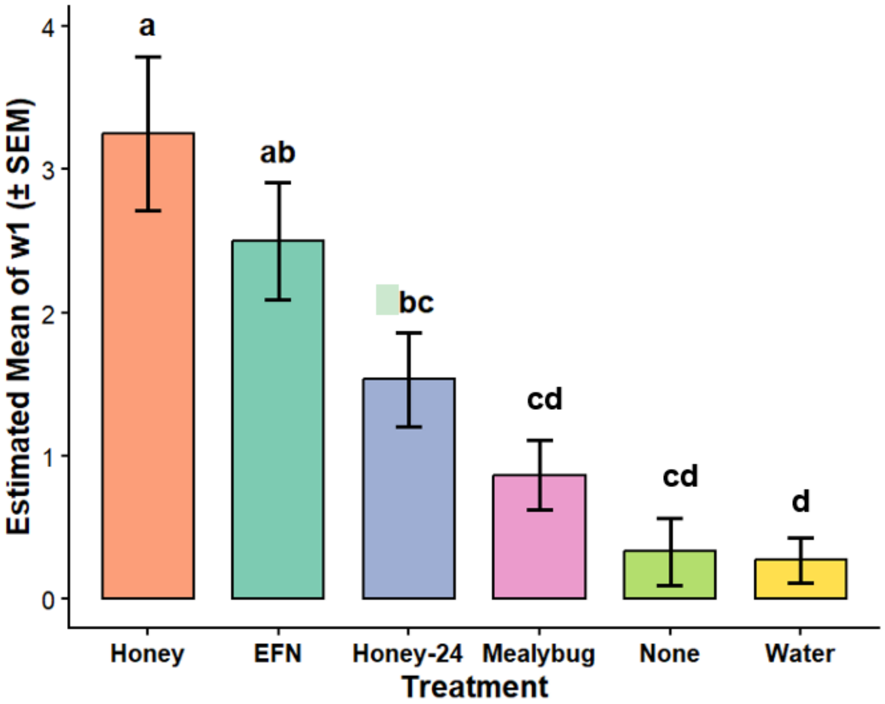

Figure S5. *Mastrus ridens* males foraging in colonies of *E. lanigerum*, and deposition of wax on its body limiting its mobility

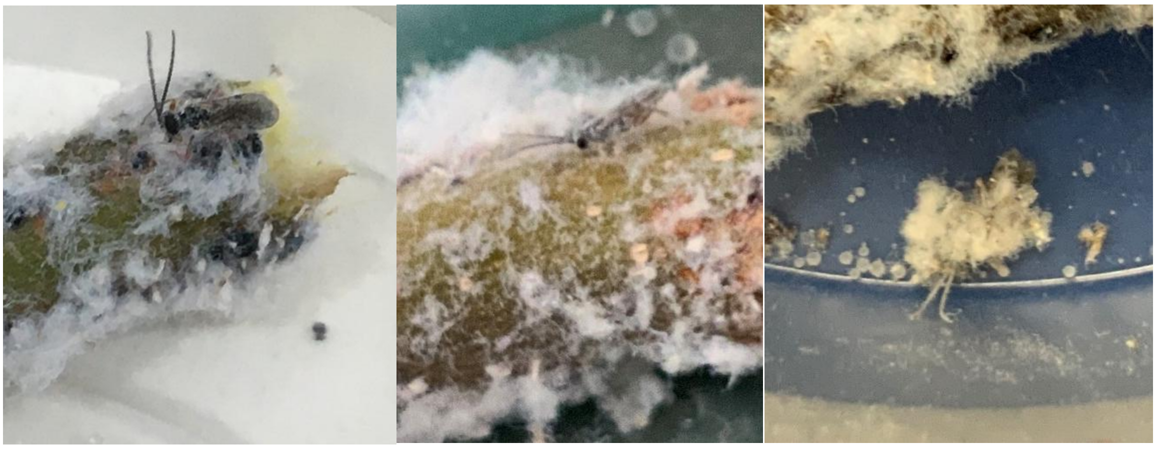

Supplement: Supplementary file 1 [file insects-17-00693-s001.zip › insects-4379576-supplementary.pdf]
